# Supplementary material for: Protective Effect of Extra Virgin Olive Oil on Cancers, Gastrointestinal Cancers, and All-Cause Mortality: A Competing Risk Analysis in a Southern Italian Cohort
Source: Cancers (Basel). 2024 Oct 23;16(21):3575. doi: 10.3390/cancers16213575 (PMC11545239; doi:10.3390/cancers16213575)
Supplement: Supplementary file 1 [file cancers-16-03575-s001.zip › cancers-3239892-supplementary.pdf]

Supplementary Materials

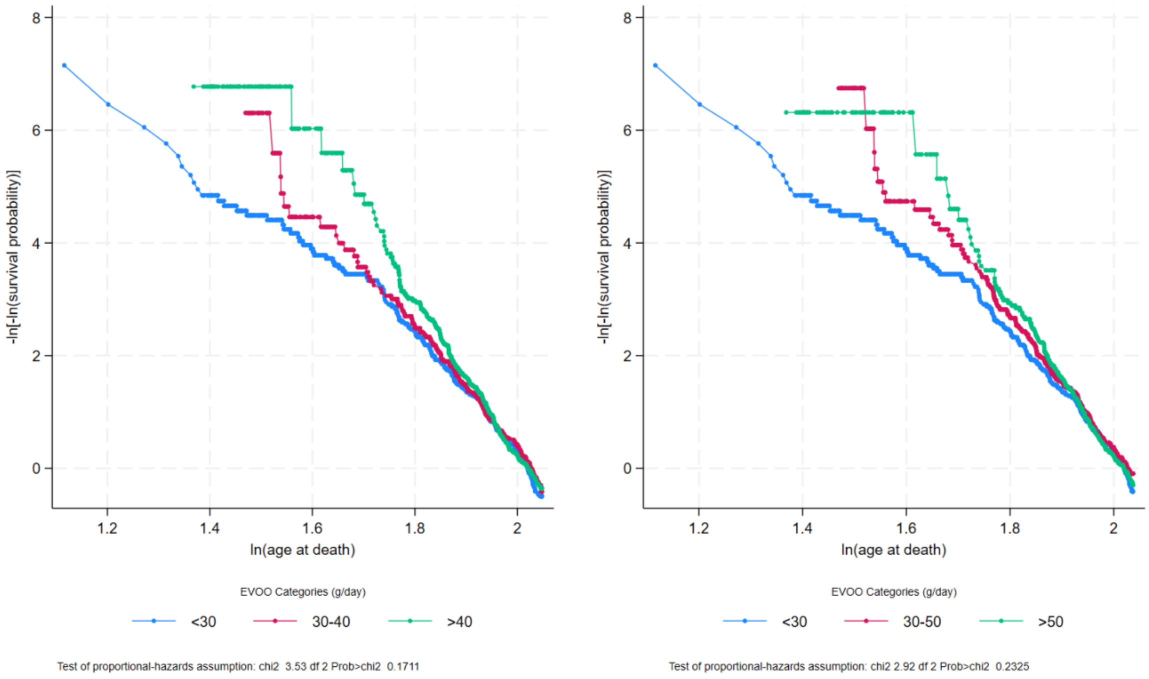

Figure S1. Tests of proportional-hazards assumption after Cox models

Table S1. Characteristics of Participants by EVOO Categories (g/day) MICOL/PANEL study. Castellana Grotte (BA), Italy, 2005-2023

	EVOO Categories (g/day)			EVOO Categories (g/day)		
	<30	30-40	>40	<30	30-50	>50
	Mean±(SD).	Mean±(SD).	Mean±(SD).	Mean±(SD).	Mean±(SD).	Mean±(SD).
Age at enrollment (yrs)	49.24 (13.80)	56.25 (14.78)	61.71 (13.53)	49.24 (13.80)	57.16 (14.46)	63.37 (13.16)
Age at death (years)	65.87 (12.37)	72.16 (12.92)	77.05 (11.82)	65.87 (12.37)	73.12 (12.71)	78.30 (11.45)
SBP (mmHg)	119.01 (18.74)	125.16 (19.57)	129.25 (19.89)	119.01 (18.74)	126.18 (20.04)	129.96 (19.34)
DBP (mmHg)	74.15 (10.52)	75.40 (9.90)	75.33 (9.65)	74.15 (10.52)	75.29 (9.88)	75.47 (9.54)
Weight (kg)	74.58 (15.11)	75.42 (15.08)	75.80 (15.56)	74.58 (15.11)	75.35 (15.06)	76.13 (15.86)
BMI (kg/m^2)	28.03 (4.91)	28.95 (5.15)	29.56 (5.42)	28.03 (4.91)	28.89 (5.04)	30.00 (5.69)
Waist (cm)	90.93 (13.41)	93.32 (12.43)	95.79 (13.25)	90.93 (13.41)	93.61 (12.35)	96.74 (13.73)
Calories days	2100.22 (722.81)	2197.98 (692.15)	2349.50 (688.76)	2100.22 (722.81)	2217.84 (688.80)	2404.53 (687.21)
Calories no Oil days	1938.85 (721.37)	1918.53 (693.49)	1885.21 (669.25)	1938.85 (721.37)	1912.65 (689.39)	1875.61 (661.76)
TG (mmol/L)	127.53 (99.75)	131.01 (91.14)	129.77 (80.57)	127.53 (99.75)	131.46 (88.73)	128.30 (78.41)
TC (mmol/L)	199.46 (38.67)	200.56 (38.54)	198.83 (38.35)	199.46 (38.67)	200.59 (38.00)	197.77 (39.08)
HDL (mmol/L)	51.31 (14.55)	50.31 (12.98)	51.60 (13.74)	51.31 (14.55)	50.57 (13.11)	51.93 (13.97)
LDL (mmol/L)	122.92 (33.50)	124.14 (33.05)	120.92 (32.86)	122.92 (33.50)	123.64 (33.01)	119.86 (32.78)
Glucose (mmol/L)	107.79 (25.99)	109.55 (28.49)	110.90 (27.17)	107.79 (25.99)	110.03 (27.87)	110.89 (27.45)
ALT (U/L)	17.58 (13.42)	17.83 (14.75)	17.85 (18.18)	17.58 (13.42)	17.83 (14.96)	17.87 (19.64)
AST (U/L)	12.24 (6.82)	13.02 (8.93)	13.81 (13.05)	12.24 (6.82)	13.13 (10.05)	14.10 (13.70)

DBP: Diastolic Blood Pressure; SBP: Systolic Blood Pressure; BMI: Body Mass Index; WHR: Waist to hip ratio; TG: Triglycerides; TC: Total Cholesterol; HDL: High-Density Lipoprotein Cholesterol; LDL: Low-Density Lipoprotein Cholesterol; ALT: Alanine Aminotransferase; AST: Aspartate Transaminase.

Table S2. Distribution of food groups consumption by Status (Alive o Causes of Death)

	Causes of Death				
	Alive	GC	OCr	DOC	*p-value*[¥]
N	2052	75	116	502	
Age at death (years)	67.31 (12.30)	77.81 (9.59)	74.76 (11.60)	83.07 (9.33)	<0.001
EVOO (g/die)	31.96 (18.42)	38.16 (22.23)	37.56 (17.73)	43.23 (19.90)	<0.001
Cereals (g/die)	280.08 (143.09)	248.33 (118.22)	251.47 (134.22)	239.20 (124.68)	<0.001
Vegetables (g/die)	250.85 (202.61)	231.41 (174.73)	266.76 (306.64)	237.84 (178.47)	0.37
Legumes (g/die)	33.15 (27.02)	30.51 (16.72)	37.83 (72.13)	33.20 (27.31)	0.34
Fish (g/die)	37.93 (29.60)	34.83 (24.65)	36.80 (35.54)	36.10 (36.49)	0.57
Total Meat (g/die)	84.09 (53.30)	72.45 (42.93)	75.50 (49.48)	63.69 (43.13)	<0.001
Dairy Products (g/die)	207.13 (148.93)	224.45 (214.40)	199.21 (126.33)	209.64 (149.78)	0.70
Fruits (g/die)	678.62 (554.45)	622.06 (545.53)	625.83 (508.44)	672.35 (569.46)	0.64
Vino (ml/die)	136.89 (196.78)	156.69 (208.56)	162.99 (182.26)	148.86 (188.95)	0.30
Total Alcohol (ml/die)	174.57 (241.96)	191.97 (256.60)	223.17 (266.30)	170.21 (228.52)	0.16

Cereals: Barley, Dry Pasta, Rice, Risottos, Pastina, Pizza, Focaccia, Bread, Biscuits, Pastries. **Vegetables**: Aubergines, Courgettes, Spinach, Cabbage, Cauliflower, Turnip Greens, Cucumbers, Watermelons, Green Beans, Fava Beans with **Vegetables**, Carrots, Artichokes, Peppers, Fennel, Celery, Swiss Chard, Chicory, Green Salad. Salad Tomatoes. **Legumes**: Broad Beans, Chickpeas, Beans, Lentils and Peas. **Fish**: Octopus, Cuttlefish, Squid, Shrimps, Anchovies, Sardines, Mussels, Mullet, Mackerel. **Total Meat**: Veal, Lamb, Horse, Rabbit, Chicken, Liver, Pork, Sausage, Mortadella, Cooked Ham, Raw Ham, Salami**. Dairy Products**: Whole Milk, Partially Skimmed Milk, Skimmed Milk, Yogurt,Grana, Parmesan, Caciottina, Stracchino, Fontina, Pecorino, Vacchino, Provolone, Caciocavallo, Formaggino, Belpaese, Gorgonzola, Scamorza, Swiss, Mozzarella, Ricotta. **Fruit**: Peaches, Watermelon, Apricots, Cherries, Grapes, White Melon, Apples, Pears, Citrus Fruits And Kiwis.

rMED: relative Mediterranean Scoring System. [¥]Kruskal-Wallis test. GC: Gastrointestinal Cancer; OCr: Other Cancer Deaths; DOC: Deaths from Other Causes.

Table S3. Subdistribution Hazard Ratios (SHR) for Mortality by EVOO Consumption Categories (g/day).

	GC	OCr
EVOO Categories (g/day)	SHR (95% CI)	SHR (95% CI)
Model 1		
<30	1.00	1.00
30-50	0.92 (0.57; 1.50)	0.86 (0.57; 1.30)
>50	0.54* (0.31; 0.96)	0.62* (0.39; 0.99)
Model 2	SHR (95% CI)	SHR (95% CI)
<30	1.00	1.00
30-50	0.92 (0.57; 1.49)	0.86 (0.57; 1.30)
>50	0.53* (0.30; 0.94)	0.62* (0.39; 1.30)

Model 1: univariate. Model 2 corrected by sex. GC: Gastrointestinal Cancer; OCr: Other Cancer Deaths;

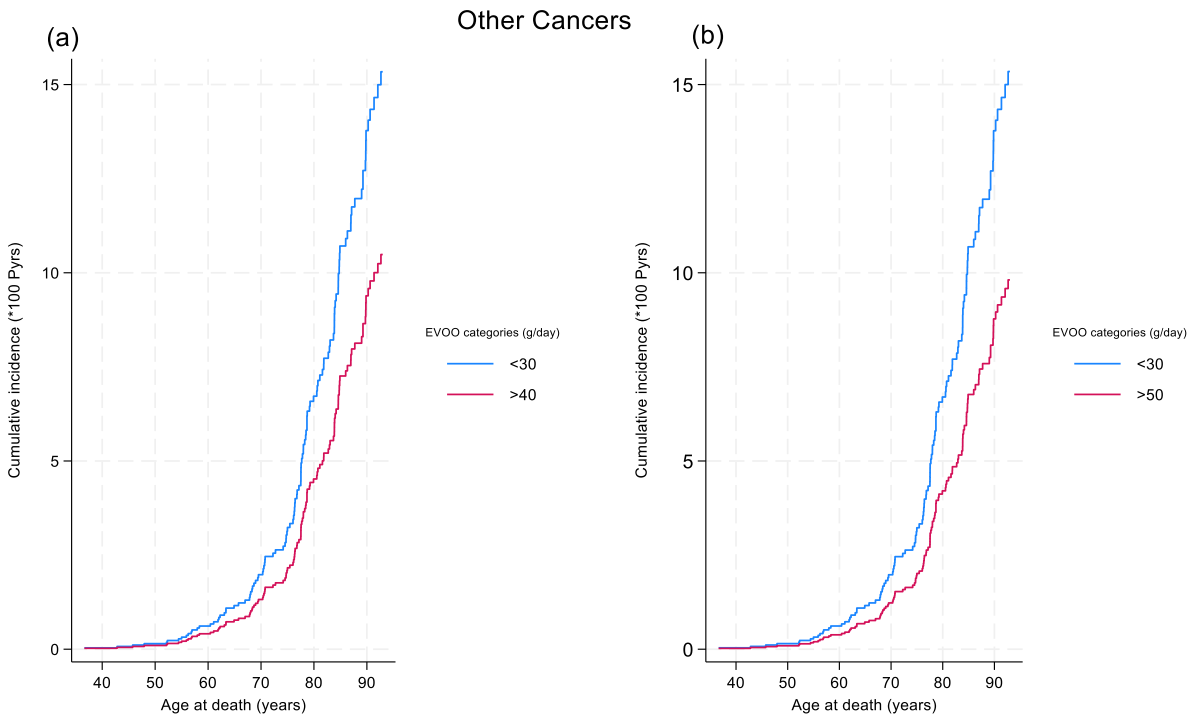

Figure S2. Comparison of Cumulative Cause-Specific Incidence Functions for Other Cancer Death by EVOO consumption categories (a) <30; (b) >40.
